# Rheumatism

**Published:** 1848-11

**Authors:** 


*Rheumatism.—Sulphate of Quinine.*—A young woman, aged twenty-three, was confined, in the month of June, at the Hospital of La Maternité. On the ninth day after parturition she left the hospital, and accidently took cold while suckling her baby at night. The consequence was, the development of an attack of acute rheumatism, which first invaded the right shoulder, and thence was propagated to the other joints. The disease had lasted six weeks with unabated violence, when the patient was admitted into the wards of Dr. Briquet, at La Charité. Eighteen grains of sulphate of quinine were prescribed, and in a few days the disease yielded to the daily exhibition of this dose.

Another case of acute articular rheumatism was admitted into M. Rayer's wards; the subject was a vigorous journeyman, aged thirty; venesection was twice performed, and ʒss. of sulphate of quinine was daily exhibited. In the space of seven days the pains had ceased, and the patient was pronounced convalescent.—*Annales de Thérapeutique.*

*Death from a Foreign Body in the Trachea.*—The subject, a corpulent and healthy-looking woman of middle age, was brought to the hospital dead, who, from the account of the bearers, had been quarrelling, and received a blow on the head, from which, after some convulsive movements, she quickly died.

When placed on the table, there was no lividity of the face, nor other peculiarity of visage; there was a superficial lacerated wound of the front of the scalp, about an inch and a half in length, and situated over the central part of the frontal suture, its anterior edge somewhat bruised, and the posterior slightly torn from the pericranium. There was slight ecchymosis around the wound, but no injury of the skull, which, indeed, was nowhere denuded of the pericranium, and on being laid open, displayed the brain and base of skull altogether uninjured. The abdominal and thoracic viscera were then laid bare, and according to the rule in examining all bodies here, whatever the nature of the case may be, the trachea was slit open, and to the surprise of all, exposed at once the cause of death. A plug of boiled saur-kraut, nearly an inch in length, was so firmly impacted in the trachea, an inch and a half below the larynx, that it must have instantly destroyed all respiration; a few strips of the vegetable were also found in the ventricles of the larynx, and the pharynx was likewise nearly full of it; the stomach contained some food, and, like the other viscera, was quite healthy.

The explanation of the case appears very simple; the body with which she had been struck must have been blunt, and, as usually happens, slipped obliquely from the skull, making a mere superficial wound, without doing further mischief there. She was, however, eating at the time, and there can be little doubt that the blow was unexpected; and, as occurs in all cases of injury and fright, it caused her to take a sudden and violent inspiration, and, in consequence, produced the sucking in of the vegetable she was about to swallow. The firm manner in which it plugged up the trachea, demonstrated the great force with which it had been drawn in, and the insufficiency of the convulsive efforts that followed to dislodge it. Had the trachea not been examined, (and how often is it neglected,) the cause of death would no doubt have been at once set down as the result of the direct effects of the blow upon the brain; and as I have not heard the issue of the legal proceedings, I cannot say how far,—if, indeed, in any degree,—the discovery of the true cause of death lightened the legal guilt of the prisoner in the eyes of the Viennese jurists.—*Lancet.*

